# Integrative evaluation of utilization the nanomaterials as a modifier to asphalt mixtures: traditional and Superpave tests

**DOI:** 10.1038/s41598-026-52794-z

**Published:** 2026-05-18

**Authors:** Mohamed Ragab, Samir Aly Kotb, Hafez Abbas Afify, Islam Abo El-Naga

**Affiliations:** 1 Civil Engineering Department, Faculty of Engineering, Suez University, Suez, Egypt; 2 Department of Public Works Engineering, Faculty of Engineering, Tanta University, Tanta, Egypt

**Keywords:** Asphalt binder, Asphalt mixtures, Marshall test, Metakaolin material, Superpave tests, Engineering, Materials science, Nanoscience and technology

## Abstract

Nanomaterials are increasingly used to improve asphalt binder and mixture performance. This research aims to evaluate the performance of asphalt binder and asphalt mixtures modified with Nano-metakaolin (NMK) using both conventional and Superpave testing methods. The experimental program conducted on the asphalt binder included penetration, softening point, flash point, and absolute viscosity tests. In addition, asphalt mixture performance was assessed through Marshall stability, indirect tensile strength, and rutting tests for both unmodified and NMK-modified mixtures. Furthermore, dynamic shear rheometer (DSR) tests, as part of the Superpave system, were conducted to determine the dynamic shear modulus (G*) of both asphalt binder and mixtures. The results indicated that the incorporation of NMK reduced penetration values, increased the softening point, and enhanced viscosity, reflecting improved binder stiffness and temperature susceptibility. Moreover, NMK significantly increased the dynamic shear modulus, indicating better resistance to deformation under loading. At the mixture level, NMK modification improved key mechanical properties, including stiffness, resistance to permanent deformation (rutting), and fracture resistance. A dosage of 7% NMK by weight of asphalt showed notable improvement in mixture strength. However, based on Superpave evaluation, the optimal NMK content ranged between 5 and 7%, providing the best balance in resistance to rutting, fatigue, and cracking. Overall, the findings demonstrate that incorporating NMK into asphalt materials enhances mechanical performance, reduces structural deterioration, and offers potential environmental benefits, making it a promising additive for advanced asphalt pavement applications.

## Introduction

When choosing a prefix for a project, many aspects must be considered, including construction ability, cost, and predicted performance. Cracking in low-temperature pavement and rutting in high-temperature pavement are caused by low-temperature fracture and high-temperature fluidity, respectively, lowering pavement quality and performance. The mixture and adhesion of bitumen and aggregate surfaces under varied situations determine the performance, serviceability, and durability of pavements. Since treating and reconstructing such defects is expensive, preventing these occurrences is a more cost-effective approach. Considering these issues, several attempts have been made to improve the durability and resistance of asphalt, as well as to enhance the quality of bitumen. Modification of bitumen properties is one of the methods for preventing damage. As a result, various bitumen modifications are employed to achieve desirable properties. For multiple purposes, including waste materials, rubber, powder materials, metakaolin, and more^1–4^. The incorporation of recycled materials into asphalt mixtures has become a common practice aimed at enhancing pavement performance. At the same time, global sustainability goals have accelerated the search for advanced and environmentally responsible materials in road construction, promoting the development of greener pavement technologies^5–7^.

Recently, nanotechnology has been applied to research to improve bitumen characteristics. According to studies in this field, using modified bitumen as a nanomaterial has gained popularity due to its high quality, versatility, and long-term performance. The production of nanocomposite materials faces many challenges, including unique mechanical and rheological characteristics of nanomaterials, as well as chemical compatibility with the bitumen matrix. One of the products of nanomaterials is nano-metakaolin, which is produced from metakaolin materials, considered one of the most important natural sources, with many uses in industry, including the production of ceramics and pottery, as well as water treatment filters. It is also used in medicine for treating the skin and removing toxins. The use of NMK in road pavement contributes to the modification of the physical properties and engineering properties of asphalt while also saving energy and money^8,9^.

Several studies have investigated the effect of nanomaterials on the performance of asphalt binders and mixtures. Santagata et al.^10^ reported that the incorporation of carbon nanotubes at dosages of 0.1% and 0.5% by weight of asphalt significantly enhanced rheological properties. Superpave test results indicated increases in viscosity and stiffness by approximately 20%, along with improvements in rutting resistance (18%), fatigue resistance (19%), aging resistance (17%), and thermal cracking resistance (22%), with the optimum performance achieved at 0.5%.

Similarly, Suzsan et al.^11^ highlighted that various nanomaterials, including nano-clay, nano-silica, and nanotubes, contribute to improved viscosity and enhanced resistance to rutting and fatigue. Diab et al.^12^ demonstrated that the addition of nano-hydrated lime (NHL) improves asphalt binder performance, increasing rutting resistance (20%), fatigue resistance (19%), thermal cracking resistance (16%), and creep stiffness (16%) compared to foamed asphalt.

Murano et al.^13^ investigated the use of metakaolin as a partial filler replacement and found that it improved Marshall stability by 25% and reduced flow by 20%, with optimal results at 20% replacement and 5.5% asphalt content. Jahromi et al.^14^ also confirmed that carbon nanofibers enhance mixture performance, increasing stability (23%), reducing flow (20%), and improving both fatigue and rutting resistance by approximately 18%.

Zhang et al.^15^ studied nano-calcium carbonate combined with polyethylene and observed improved stiffness, softening point, and flexibility compared to polyethylene-modified binders alone. In addition, Hamedi et al.^16^ showed that nanoparticle coatings (iron oxide and aluminum oxide) significantly reduce moisture susceptibility, enhance adhesion, and decrease stripping potential by about 20%, with iron oxide showing superior performance.

Blom et al.^17^ reported that nano-clay improves softening point (20%), stiffness (8%), rutting resistance (18%), and fatigue resistance (14%), although it slightly reduces adhesion. Furthermore, Abo El-Naga and Ragab^18^ demonstrated that recycled PET significantly enhances asphalt performance by increasing stiffness, improving resistance to rutting and cracking, and extending pavement service life.

Overall, these studies confirm that nanomaterials effectively enhance the mechanical, rheological, and durability properties of asphalt mixtures. However, the performance improvements strongly depend on the type and dosage of the nanomaterial, with optimal results generally achieved at moderate additive contents. Despite the extensive research on nanomaterials in asphalt modification, limited studies have focused on Nano-metakaolin (NMK), particularly in a comprehensive manner that evaluates both asphalt binder and mixture performance. In addition, the combined use of conventional and Superpave testing methods to assess NMK over a wide range of dosages remains insufficiently addressed. Therefore, this study aims to fill this gap by investigating the effect of NMK on asphalt binder and mixtures using an integrated experimental approach.

## Research methodology

This study investigates the effectiveness of Nano-metakaolin (NMK) as a modifier for asphalt binders and asphalt mixtures through a comprehensive experimental program. The research methodology is structured into two main phases, as illustrated in the study framework. In the first phase, a laboratory testing program was conducted to evaluate the effect of NMK on asphalt binder properties. NMK was incorporated at dosages of 3%, 5%, 7%, 9%, and 11% by weight of asphalt. A series of conventional tests, including penetration, softening point, flash point, and kinematic viscosity, were performed. In addition, Superpave-based testing using the dynamic shear rheometer (DSR) was carried out to determine the rheological properties of both unmodified and NMK-modified binders. This phase aimed to characterize the fundamental physical and rheological behavior of the modified asphalt binders. In the second phase, NMK was incorporated into asphalt mixtures at proportions of 3%, 7%, 9%, and 11% to evaluate its impact on mixture performance. Mechanical and performance-related tests, including Marshall stability, indirect tensile strength, wheel tracking, and complex shear modulus tests, were conducted. This phase focused on assessing the key mechanical properties of the modified asphalt mixtures, particularly in terms of stiffness, deformation resistance, and cracking performance.

## Qualification tests and materials

### Components of asphalt mixes

A single supplier was selected for each component of the asphalt mixture to achieve the objectives of this inquiry and to mitigate the impact of their fluctuations on the quality of the asphalt mixture. A fine aggregate from the “FAYED” quarry, procured by the US government, was utilized in this blend. Table 1 presents the gradation of fine aggregate, whereas Table 2 delineates its features.

**Table 1:** The fine Aggregate used and gradation.

The sieve size (mm)	% of passing
25	100
19	100
12.7	99.5
9.52	82.7
4.75	15.5
2.36	8.7
0.60	6.5
0.30	4.3
0.15	3
0.075	1.6

**Table 2:** Characteristics of fine aggregates.

Test	Designation by AASHTO No.	The end result	Specification restrictions
Bulk specific gravity		2.65	-
Dry surface of saturated		2.66	-
Apparent SG		2.64	-
	T-85		-
			-
			-
Absorption of water (percent)	T-85	1.71	Less or equal 5

The coarse aggregate consisted of crushed siliceous dolomite sourced from the “ATAKA” quarry in Egypt’s Suez Governorate. Tables 3 and 4 present the coarse aggregate and its attributes, respectively. Table 5 presents the gradation of the planned aggregates alongside the relevant specification limits for the examined mixes. Table 6 delineates the characteristics of the bituminous materials utilized in this investigation, sourced from the Suez Petroleum Company.

**Table 3:** The gradation of used coarse aggregate.

Sieve size (mm)	% passing	
	Size 1.5 inch	Size 2 inch
25	100	100
19	24.4	50.6
12.7	1.6	0.9
9.52	0.7	0.3
4.75	0.5	-
2.36	-	-

**Table 4:** The characteristics of used coarse aggregates.

Test	AASHTO specs	Results	Specification limits
Bulk specific gravity	T-85	2.526	-
Saturated surface dry specific gravity		2.538	-
Apparent specific gravity		2.65	-
Absorption of water%	T-85	2.538	Less or equal 5
Disintegration%	T-112	0.72	Less or equal 1
Abrasion% afer 100 rev	T-96	6.5	Less or equal 10
Abrasion% after 500 rev		26	Less or equal 40
Stripping%	T-182	Less than 95	Less or equal 95

**Table 5:** The characteristics of the used aggregate in use for the investigated mixture.

Sieve size (mm)	Designed gradation	Design limits
25.4	100	100
19.1	75.8	70–100
12.7	64.2	60–90
9.52	57.2	50–80
4.75	30.2	30–65
2.36	25.6	25 -50
0.60	16.5	15–30
0.30	9.5	10–25
0.15	3.6	3–10
0.075	1.7	0–3

**Table 6:** The properties of used asphalt binder.

Test	AASHTO specs	Results	60/70 Bitumen specification
Penetration (0.1 mm)	T-45	66	60–70
Softening point (°C)	T-53	50	45–55
Flash point (°C)	T-48	250	+ 250
Kinematic viscosity (Cst)	T-201	426	+ 320

### Nano-metakaolin (NMK) material

**Fig. 1:**
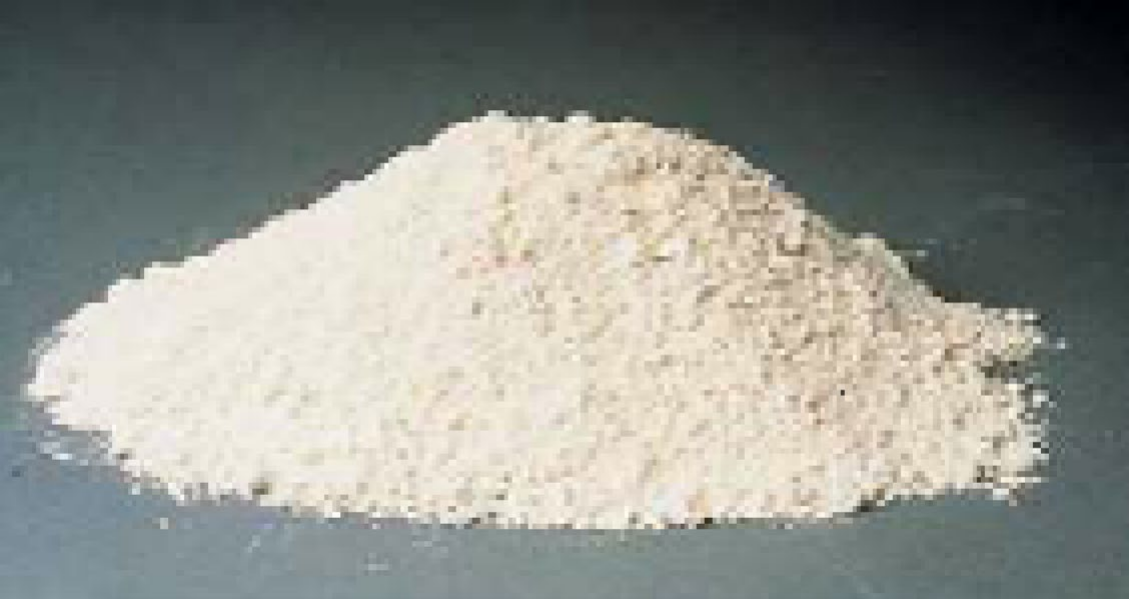
Preparation of nano-metakaolin (NMK).

Nano-metakaolin (NMK) is a mineral admixture produced through the thermal treatment of kaolin, where calcination is applied to obtain reactive metakaolin, followed by high-energy ball milling to achieve nano-sized powder particles, as shown in Fig. 1. This material is considered suitable for asphalt modification due to its relatively high thermal stability (up to 150–175 °C), as well as its excellent mechanical strength, hardness, and thermal resistance. In addition, NMK exhibits good resistance to hydrolysis, chemical attack, and solvent effects, attributed to its rigid structure.

**Fig. 2:**
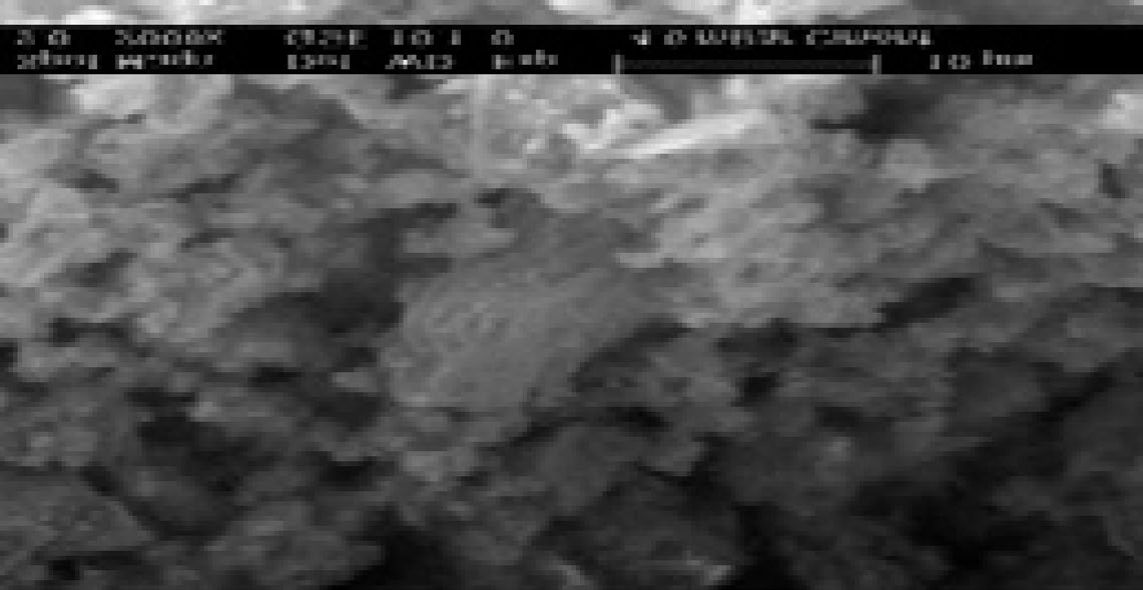
XRD analysis of NMK.

The NMK used in this study is a locally available material obtained from Shak El-Thoaban area, Cairo, Egypt, without further chemical treatment. It has a bulk density of approximately 2.66 g/cm^3^, a specific surface area ranging from 8–15 m^2^/g, and a brightness index of 80–82 Hunter units. X-ray analysis was conducted at the Central Laboratory of Tanta University to verify the physicochemical characteristics of the material and confirm its transformation into nano-sized particles, as shown in Fig. 2. The results confirmed that the material complies with relevant specifications, as presented in Table 7.

**Table 7:** Physical characteristics of NMK.

Description	Property
Physical form	Powder
color	Off white–gray to buff
Brightness	80–82 Hunter L
Bet	15 m^2^/gram
Specific gravity	2.4–2.6
Shape of particles	cubic
Specific surface	8–15 m^2^/gram
Average nano size	40 nm
Formula sum	O_2_ Si
Oxide composition	Silicon oxide-Alpha quartz and other unknown material
Density	2.660 g/cm^3^

### Sample preparation

#### Modified asphalt binder

Nano-metakaolin (NMK) was preheated to 140 °C in a controlled container to ensure uniform thermal conditioning prior to mixing. The asphalt binder was separately heated to 140 °C in an oven until it reached a fully fluid state. NMK was then gradually incorporated into the hot binder under continuous mechanical stirring at a constant temperature. The mixing process was maintained for a specified duration to achieve complete dispersion of NMK particles and to ensure the formation of a homogeneous modified asphalt binder suitable for mixture preparation^19^.

#### Modified mixture

Nano-metakaolin (NMK) was incorporated into the asphalt mixtures through a dry mixing process with the aggregates. The aggregates were first heated to 250 °C for approximately two hours to ensure proper thermal conditioning. Subsequently, the heated aggregates were blended with NMK, which had been preheated to 140 °C, prior to the addition of the asphalt binder to produce NMK-modified asphalt mixtures^20^.

## Laboratory testing program

### Modified asphalt binder program

The laboratory testing program involved penetration, softening point, flash point, and absolute viscosity tests performed on both unmodified and modified asphalt binders in order to assess the influence of the modifier on their properties. Various quantities of NMK content are utilized to produce modified asphalt cement based on the weight of asphalt. The percentages are 1%, 3%, 5%, 7%, 9%, and 11%. The modifier is incorporated into the asphalt binder for 2 h at 140 °C to produce modified asphalt. The modifier amounts are selected based on previous literature and preliminary trials and blended with modified asphalt for two hours to meticulously assess the range of modifier quantities that can be utilized as an enhancement material safely and economically.

#### Penetration test

The penetration test is used to grade paving bitumen and assess binder consistency at intermediate service temperatures. A 100 g needle is applied to the asphalt specimen at 25 °C for 5 s, and the penetration depth is measured in 0.1 mm units (pen), where higher values indicate a softer binder^21^.

#### Softening point test

The softening point test determines the temperature at which asphalt reaches a specified softness. A bitumen sample is placed in a metal ring and subjected to a controlled heating rate of 5 °C/min in a water bath. A steel ball is positioned on the sample, and the softening point is recorded as the temperature at which the binder deforms and allows the ball to fall 25 mm^21^.

#### Flash point test

The flash point test involves heating an asphalt binder sample in a brass cup and periodically passing a flame over its surface. The flash point is the temperature at which a brief ignition occurs, and it is specified to ensure safe handling of the binder^22^.

#### Kinematic viscosity test

Bitumen viscosity indicates its resistance to flow at a specific temperature. It is measured by drawing the sample under vacuum through a viscometer at 135 °C and recording the flow time between two marks. The viscosity is determined by comparing this time with that of a reference fluid of known viscosity^22^.

#### Dynamic shear rheometer (DSR) test

The dynamic shear rheometer (DSR) test for asphalt binders (AASHTO T315) consists of two parallel metal plates, an environmental chamber, a loading system, and a data acquisition and control unit. The DSR is used to evaluate key parameters within the Performance Grading (PG) system. For testing unmodified binders and assessing rutting potential, the test temperature is selected based on the high-temperature grade of the binder. The applied loading is controlled in terms of strain or stress amplitude, depending on the magnitude of the complex shear modulus of the binder. Specification testing is conducted at an angular frequency of 10 rad/s. The rheometer software automatically determines the complex shear modulus (G*) and phase angle (δ). Wehere G* as an indicator of the material’s resistance to shear deformation, calculated from the ratio of stress to strain amplitudes. The phase angle (δ) represents the lag between stress and strain under cyclic loading and is expressed in degrees, indicating the viscoelastic nature of the binder^21^.

### Modified asphalt mixture program

At this stage, asphalt binders and mixtures were modified using NMK at different contents of 5, 7, 9, and 11% by weight of asphalt. For the prepared mixtures, a series of tests were conducted, including Marshall stability, indirect tensile strength, and wheel tracking tests. In addition, Superpave dynamic shear rheometer (DSR) tests were performed on asphalt binders, while Superpave dynamic modulus tests were conducted on the modified asphalt mixtures, as detailed in the following subsections.

#### Marshall test

The Marshall mix design method was employed to identify the optimum asphalt content and to assess both the physical properties (air voids, voids in mineral aggregate, and unit weight) and mechanical properties (stability, flow, and Marshall stiffness modulus) of the studied mixtures. The testing procedure was conducted in accordance with AASHTO T-246^18,19^. The experimental program was divided into two stages. In the first stage, aggregate mixtures were prepared with asphalt binder contents of 3.5%, 4.5%, 5.5%, and 6.5% by weight of the total mix. In the second stage, mixtures were prepared using the optimum asphalt content of 4.6%, incorporating NMK at 0, 5, 7, 9, and 11% by weight of asphalt in the modified mixtures.

#### Indirect tensile test

The indirect tensile strength (σ_t_) indicates the cracking susceptibility of asphalt mixtures. The test was performed at 30 °C by applying a compressive load along the specimen’s vertical diameter at 0.04 in/min using steel loading strips to ensure uniform load distribution.

#### Wheel tracking test

The wheel tracking test simulates rutting in asphalt mixtures using slab specimens (44 × 33 × 5 cm) tested at 60 °C under 0.625 MPa load. A pneumatic wheel applies repeated passes at 42 passes/min for 1 h, and rut depth is recorded every 5 min. The test was developed by the British Road Research Laboratory^25–29^.

#### Complex shear modulus test

The complex shear modulus and phase angle are evaluated over a range of temperatures and loading frequencies. During the test, one of the parallel plates oscillates relative to the other at specified frequencies, inducing shear deformation in the sample. The response is measured at angular frequencies corresponding to 0.1, 0.5, 1, 5, 10, and 25 Hz. The rheometer software automatically calculates the complex shear modulus (G*) and phase angle (δ), which characterize the viscoelastic behavior of the asphalt binder.

## Analysis and results

### Influence of modification on the properties of asphalt binder

#### Traditional tests results

**Fig. 3:**
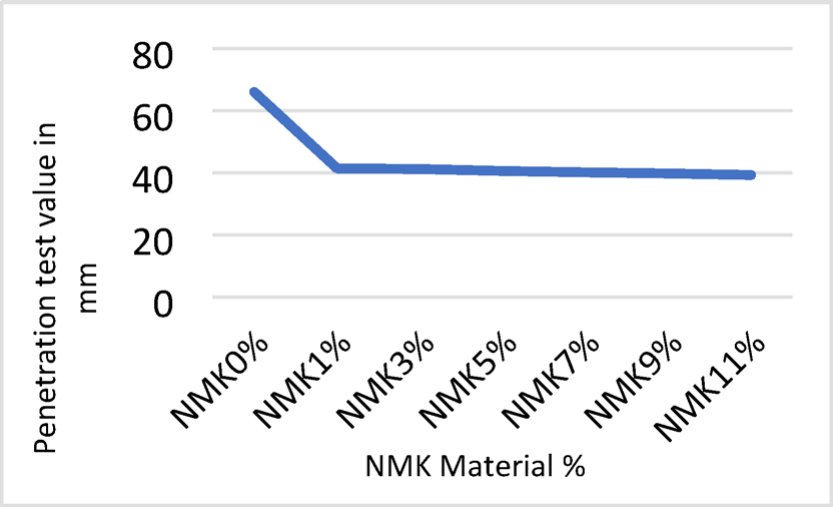
Results of Penetration test.

**Fig. 4:**
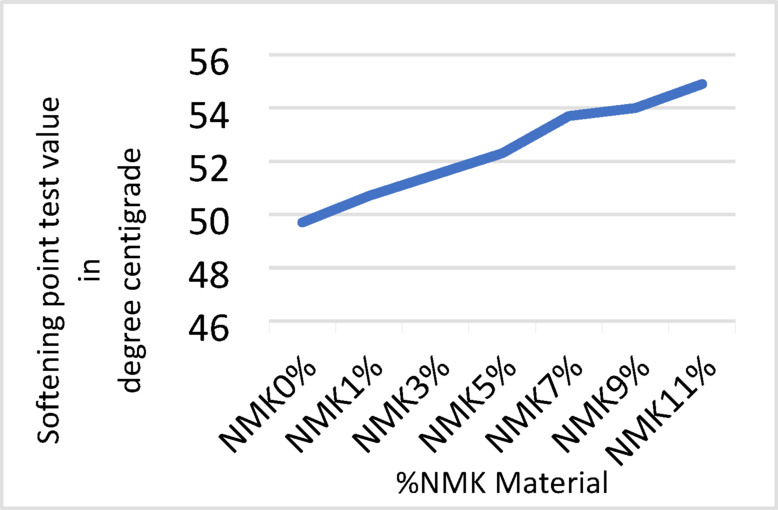
Results of softening point.

**Fig. 5:**
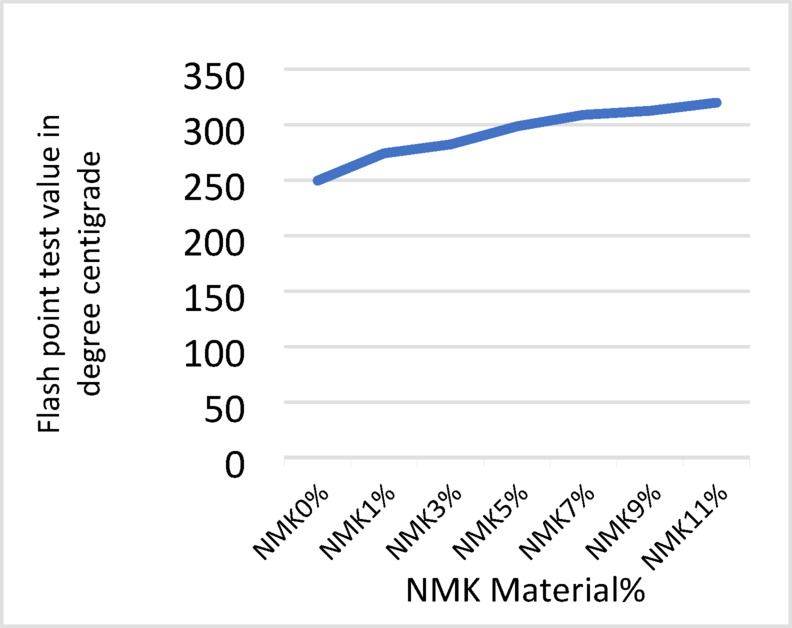
Results of flash point test.

**Fig. 6:**
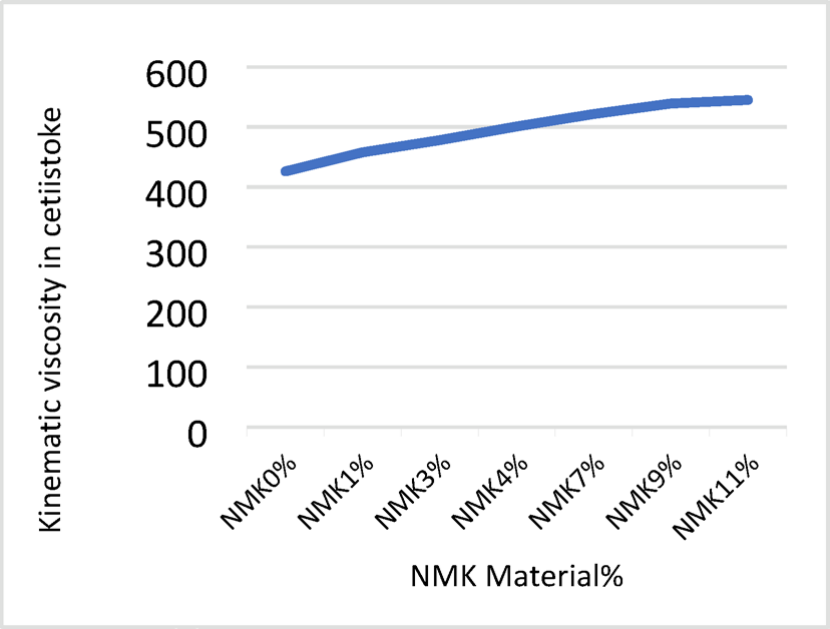
Results of kinematic viscosity test.

The results of the laboratory testing program, including penetration, softening point, flash point, and absolute viscosity tests, are presented in Tables 8, 9, 10 and 11 and illustrated in Figs. 3, 4, 5 and 6. The effect of modification on asphalt binder properties indicates that the incorporation of NMK leads to a reduction in penetration values, reflecting increased binder stiffness and reduced elasticity. The increase in softening point suggests decreased temperature susceptibility, which is beneficial in hot climates. Additionally, the inclusion of NMK enhances both the flash point and kinematic viscosity of the asphalt binder.

**Table 8:** Results of penetration test for asphalt binder.

NMK %	0%	1%	3%	5%	7%	9%	11%	Specification limit of asphalt 60–70
Penetration value of specimen (1) in 0.1 mm	67	42.5	41.8	41.3	40.3	39.9	39.1	60–70
Penetration value of specimen (2) in 0.1 mm	65	41.8	41.6	40.6	40.6	40.3	39.9	60–70
Penetration value of specimen (3) in 0.1 mm	66	39.9	40.2	39.8	39.5	39.1	38.8	60–70
Mean	66	41.4	41.2	40.57	40.13	39.77	39.27	60–70
Standard deviation%	%1	1.19%	0.87%	o.75%	0.87%	1.19%	0.79%	-

**Table 9:** Results of softening point test for asphalt binder.

NMK %	0%	1%	3%	%5	7%	9%	11%	Specification limit of asphalt 60–70
Softening point value of specimen (1) in ^o^C	50.3	50.8	51.2	52.0	53.2	54.1	54.0	45–55
Softening point value of specimen (2) in ^o^C	50	50.2	51.4	52.1	53.5	53.8	55.2	45–55
Softening point value of specimen (3) in ^o^C	49.7	50.7	51.5	52.3	53.7	54.0	54.9	45–55
Mean	50	50.57	51.37	52.14	53.47	53.97	54.7	45–55
Standard deviation%	0.3%	0.33%	0.17%	0.2%	0.26%	o.17%	0.88%	-

**Table 10:** Results of flash point test for asphalt binder.

NMK %	0%	1%	3%	5%	7%	9%	11%	Specification limit of asphalt 60–70
Flash point value of specimen (1) in ^o^C	250	275	283	295	309	311	320	250 or more
Flash point value of specimen (2) in ^o^C )	249	277	281	300	310	314	321	250 or more
Flash point value of specimen (3) in ^o^C	250	271	283	301	308	313	319	250 or more
Mean	249.67	274.34	282.34	298.67	309	312.67	320	250 or more
Standard deviation%	0.58%	3.06%	1.16%	3.22%	1%	1.53%	%1	-

**Table 11:** Results of kinematic viscosity test for asphalt binder.

NMK %	0%	1%	3%	5%	7%	9%	11%	Specification limit of asphalt 60–70
Kinematic Viscosity value of specimen (1) in Cst	424	452	479	500.9	523.2	540	545	320 or more
Kinematic Viscosity value of specimen (2) in Cst	426	455	475	500.4	520.3	539	544	320 or more
Kinematic Viscosity value of specimen (3) in Cst	428	457	480	501.1	521.1	538	546	320 ore more
Mean	426	454.67	478	500.8	521.54	539	545	320 or more
Standard deviation%	2%	2.52%	2.65%	0.36%	1.5%	1%	1%	-

#### Dynamic shear rheometer (DSR) test results

**Fig. 7:**
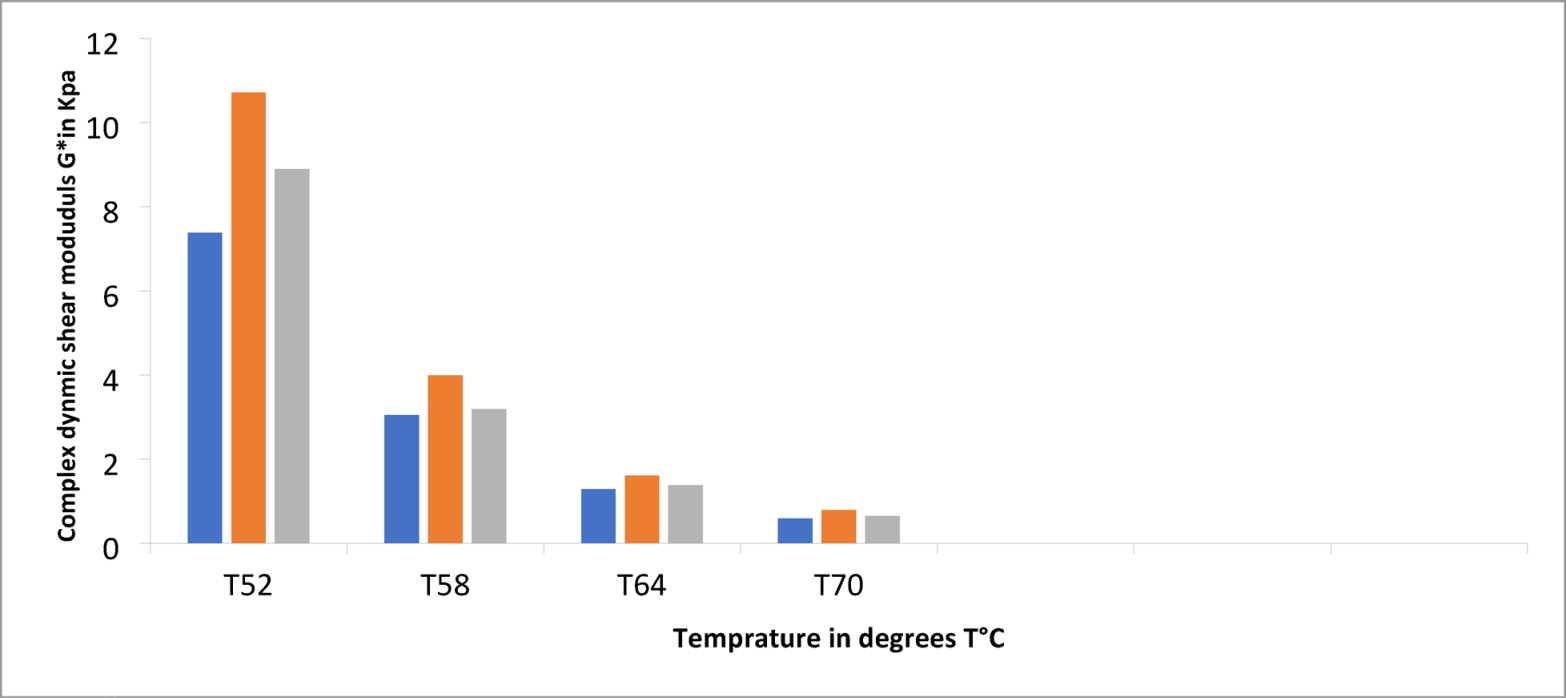
Relation between temperature and complex dynamic modulus with different percentage of NMK for asphalt binder.

This test was conducted on both unmodified and modified asphalt binder samples. The complex shear modulus (G*) and phase angle (δ) were used to characterize the resistance of the binders to shear deformation. The results are presented in Table 12 and illustrated in Fig. 13. The expriments were performed at temperatures of 52, 58, 64, and 70 °C. The results indicate that the addition of NMK improves the rheological properties of both modified and unmodified binders at high temperatures. Specifically, G* increases with NMK content up to 5% compared to the control binder (0% NMK), indicating enhanced rutting resistance. However, further increasing the NMK content to 7% leads to a reduction in G*, particularly at higher temperatures. At 70 °C, the binder exhibits reduced performance, indicating increased susceptibility to rutting, fatigue, and cracking. Overall, the optimal performance was achieved at 5% NMK content, as shown in Table 12 and Fig. 7.

**Table 12:** Dynamic Modulus Test Results for unmodified and modified asphalt binder.

Temperature (°C)	Modified asphalt binder			Unmodified asphalt binder					
				5%			7%		
	Modulus, KPa G*	Phase angle, Degree δ	Shear modulus (G*/sin δ)	modulus, KPa G*	Phase angle, Degree δ	Shear modulus (G*/sin δ)	modulus, KPa G*	Phase angle, Degree δ	Shear modulus (G*/sin δ)
52	7.3068	81.03°	7.379 ≥ 1 Pass	10.4987	78.55°	10.7119 ≥ 1 Pass	8.7592	79.94°	8.8959 ≥ 1 Pass
58	3.0274	83.35°	3.0479 ≤ 1 Pass	3.9493	81.78°	3.9903 ≥ 1 Pass	3.1656	82.92°	8.8959 ≥ 1 Pass
64	1.2802	85.41°	1.2843 ≤ 1 Pass	1.6895	84.27°	1.6979 ≥ 1 Pass	1.3736	85.07°	1.3787 ≥ 1 Pass
70	0.5921	87.00°	0.5921 ≥ 1 fail	0.7868	86.14°	0.7886 ≥ 1fail	0.6464	86.67°	0.6475 ≥ 1 fail

### Influence of modification on the properties of asphalt mixtures

#### Marshall test results

**Fig. 8:**
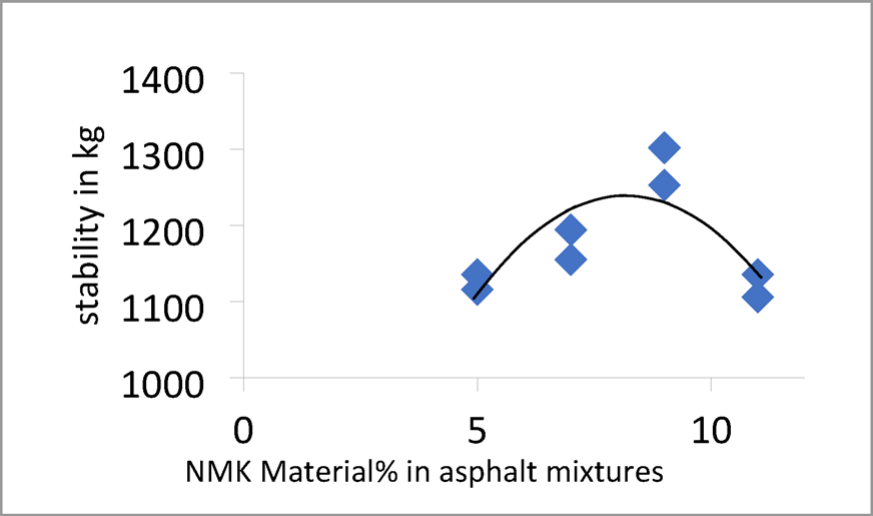
Stability versus NMK %

**Fig. 9:**
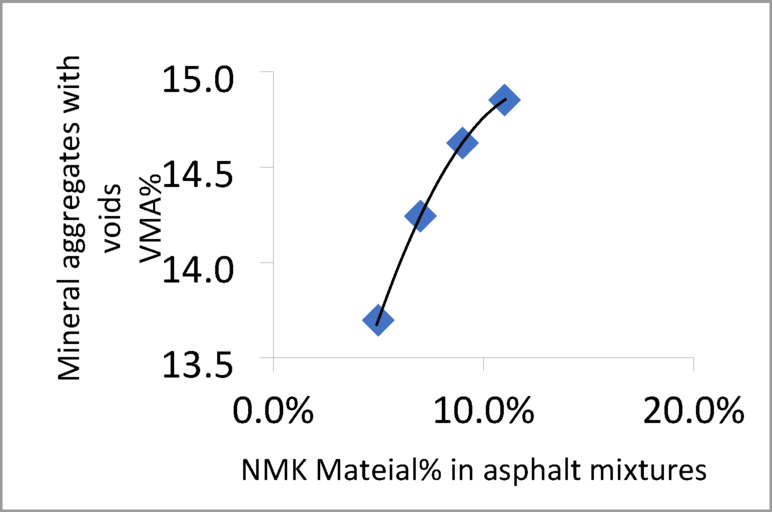
Air Voids versus NMK %

**Fig. 10:**
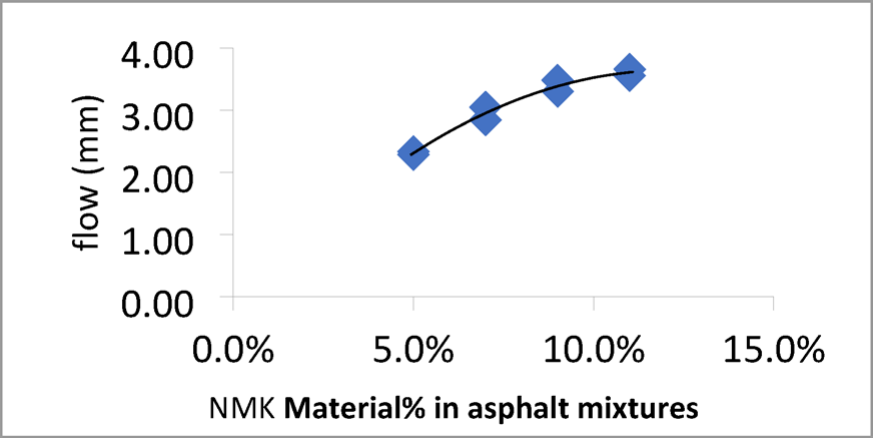
Flow versus NMK %

**Fig. 11:**
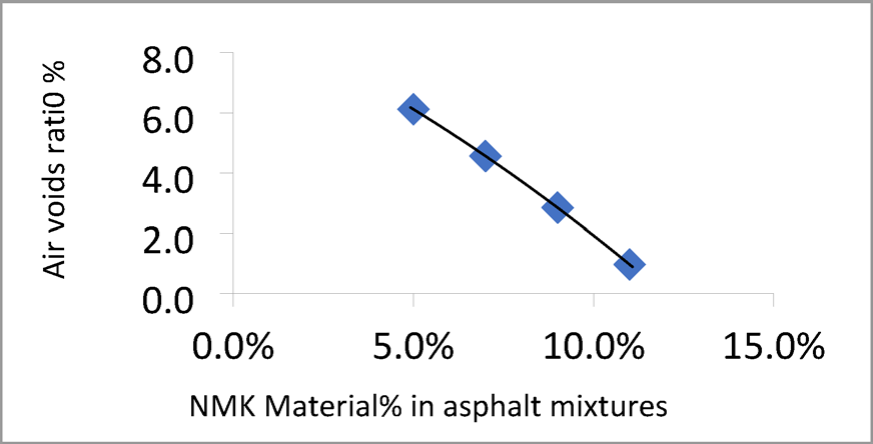
Air Voids in mineral aggregates versus NMK %

**Fig. 12:**
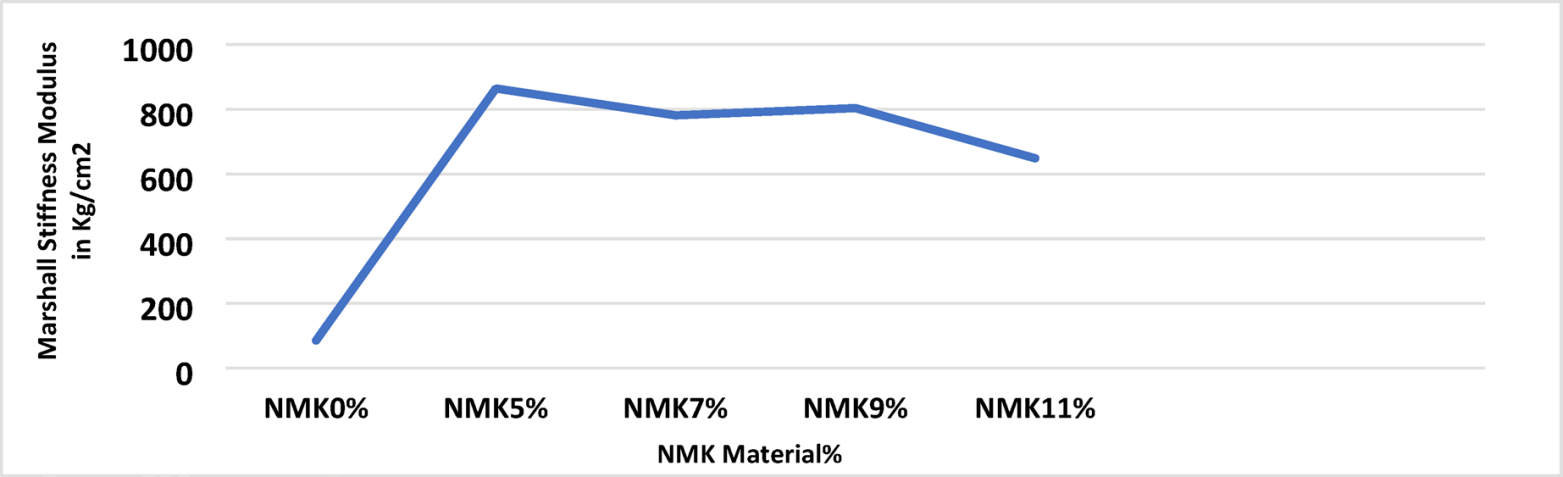
Relation between the percentage of NMK material in asphalt mixtures and Marshall stiffness.

The Marshall test results for mixtures containing different percentages of NMK in the asphalt binder are presented in Table 13 and illustrated in Figs. 8, 9, 10 and 11. Figure 8 shows the relationship between NMK content and the stability of asphalt mixtures, indicating that stability increases with higher NMK content. Figure 9 illustrates the relationship between NMK percentage and air voids, showing that air voids increase as NMK content increases. This may be attributed to the interaction of swollen NMK particles, which promote particle aggregation and reduce workability during compaction, resulting in higher void content. Figure 10 presents the variation of flow values with different NMK percentages for the studied mixtures. Figure 11 shows the relationship between NMK content and voids in mineral aggregate (VMA), indicating an increasing trend with higher NMK concentrations. This behavior may be explained by the influence of compaction characteristics, since the same factors affecting compaction also govern VMA values. Figure 12 shows the relationship between the percentage of NMK material and Marshall stiffness.

**Table 13:** Results of the Marshall Test corresponding mixture Characteristics.

NMK %	AC%	γ gm/ cm^3^	Stability kg	Flow mm	AV%	VMA%	M*s kg/cm^2^	IT kg/cm^3^**
0	4.9	2.217	1052.5	2.975	6.5	16	598.19	2.75
5	4.6	2.287	1126	2.315	6.1	13.7	879.85	3.50
7	4.6	2.294	1174.5	2.945	4.6	14.2	1025.49	3.69
9	4.6	2.306	1279.5	3.57	2.8	14.6	1138.55	3.97
11	4.6	2.322	1194.5	3.61	1.0	14.9	648.29	3.45

*MS: Marshall Stiffness modulus = stability (Flow × Specimen Thickness).

**It: Indirect Tensile Strength.

#### Indirect tensile strength test results

**Fig. 13:**
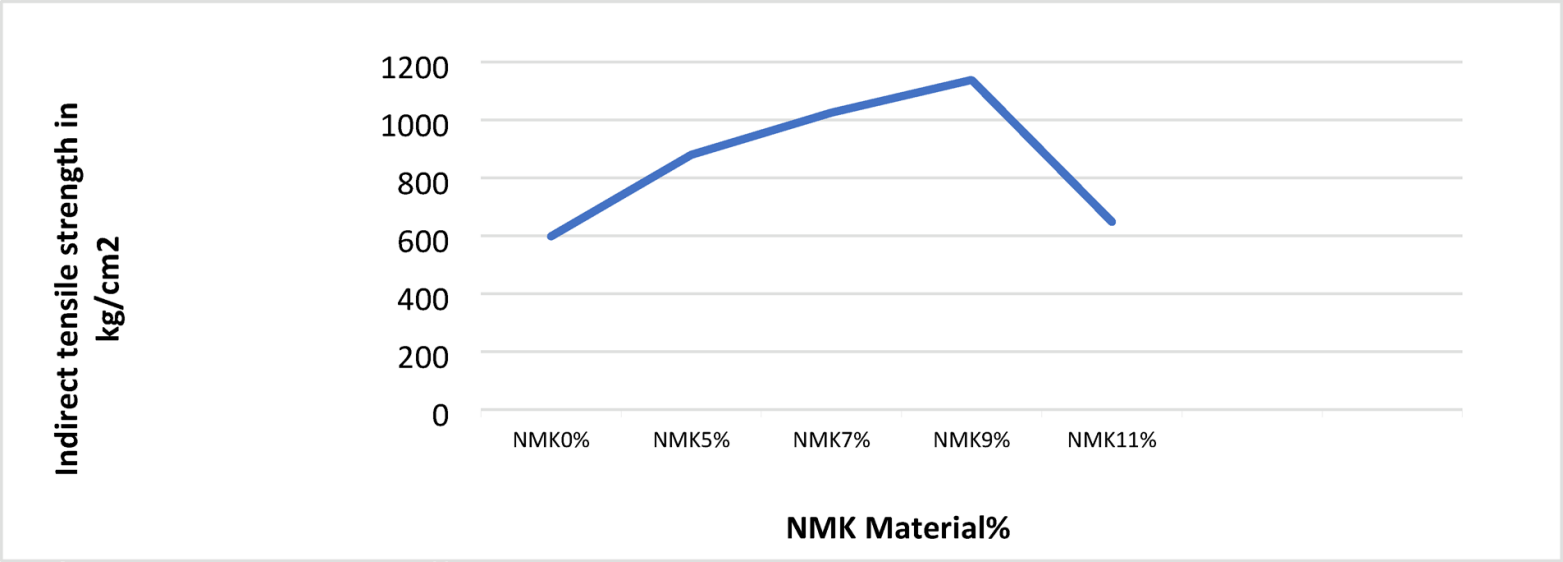
Relation between the percentage of NMK Material in asphalt mixtures and the indirect tensile strength.

The correlation between indirect tensile strength (ITS) and NMK content for the investigated mixtures is shown in Table 13 and Fig. 13. The ITS increases with increasing NMK content up to 9%, and then decreases at higher NMK contents.

#### Wheel tracking test results

**Fig. 14:**
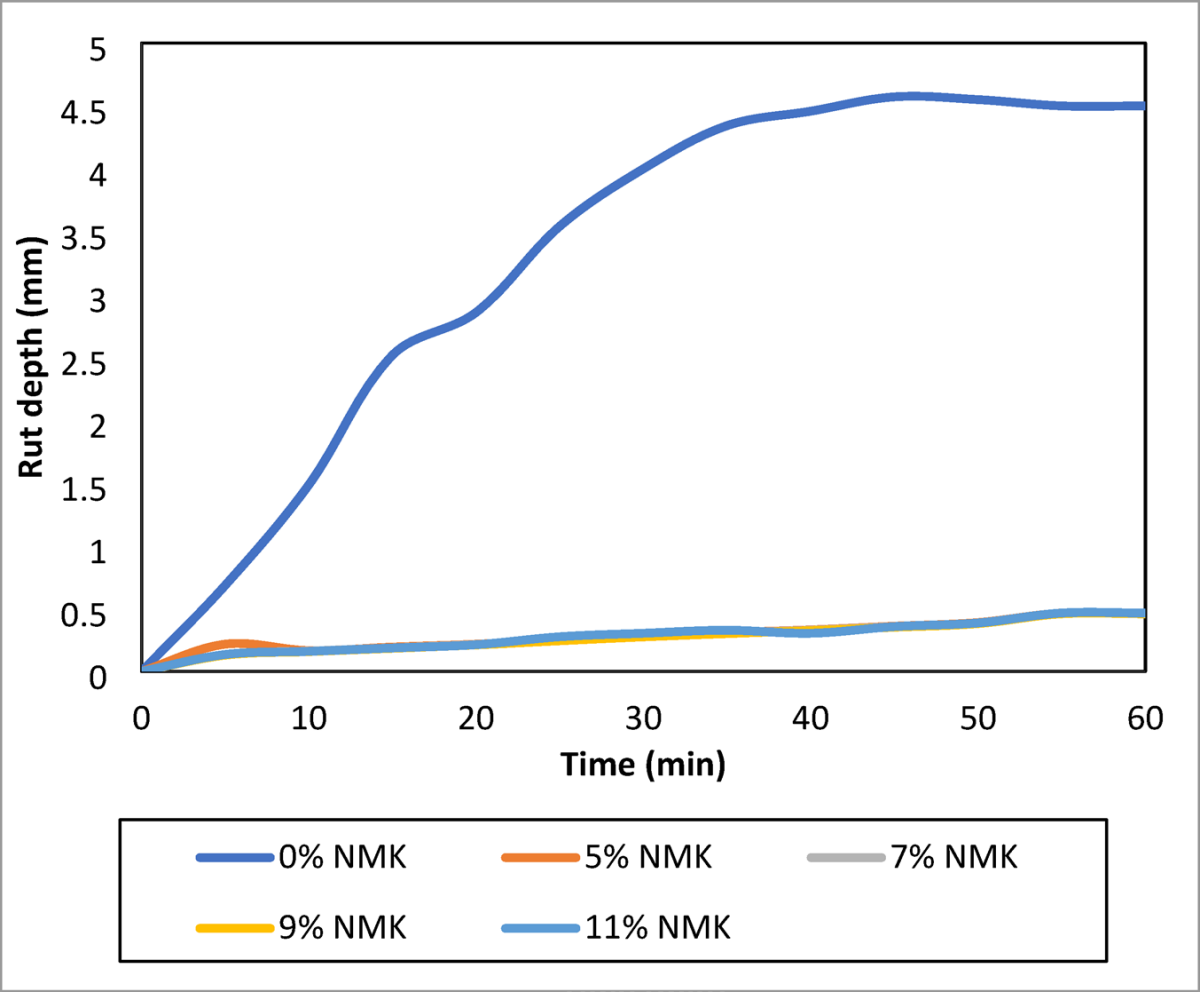
Relationship between the percent ofNMK Material and Rut depth.

The relationship between rut depth and asphalt mixtures containing different NMK percentages is presented in Table 14 and illustrated in Fig. 14. The results indicate that all NMK-modified mixtures exhibit lower rut depth over time compared to the unmodified mixture, reflecting enhanced resistance to permanent deformation. This improvement becomes more pronounced with increasing NMK content. Specifically, mixtures containing 5%, 7%, 9%, and 11% NMK demonstrate superior rutting resistance relative to the control mixture. Overall, these findings confirm that the incorporation of NMK has a significant positive effect on improving the rutting performance of asphalt pavements.

**Table 14:** Rut depth and rutting stiffness.

Time min	NMK %									
	0		5		7		9		11	
	R (mm)	Sr (kg/cm^2^)	R (mm)	Sr (kg/cm^2^)	R(mm)	Sr (kg/cm^2^)	R(mm)	Sr (kg/cm^2^)	R(mm)	Sr (kg/cm^2^)
5	0.686	184.59	0.217	924.70	0.134	945.20	0.130	970.84	0.135	938.53
10	1.486	85.09	0.166	864.28	0.162	781.20	0.158	803.61	0.160	790.02
15	2.517	58.28	0.194	651.06	0.188	672.30	0.184	685.98	0.187	780.02
20	2.858	44.28	0.217	583.47	0.214	590.85	0.211	6598.41	0.213	676.08
25	3.543	35.71	0.251	504.00	0.248	511.38	0.244	518.94	0.276	593.37
30	4.000	31.64	0.280	452.16	0.277	456.57	0.275	461.07	0.304	513.27
35	4.343	29.14	0.309	409.72	0.305	414.81	0.301	419.76	0.328	458.10
40	4.458	28.39	0.331	382.14	0.328	386.37	0.325	388.8	0.304	416.07
45	4.572	27.37	0.360	351.56	0.356	355.14	0.352	389.52	0.357	385.29
50	4.550	27.00	0.389	325.52	0.386	327.78	0.381	358.74	0.386	327.78
55	4.500	26.95	0.465	270.45	0.464	272.55	0.459	272.50	0.465	272.56
60	4.500	29.95	0.465	270.45	0.464	272.55	0.459	272.50	0.465	272.56

R: Rut Depth in (mm).

Sr: Rutting Stiffness in (kg/cm^2^).

#### Dynamic modulus test results

**Fig. 15:**
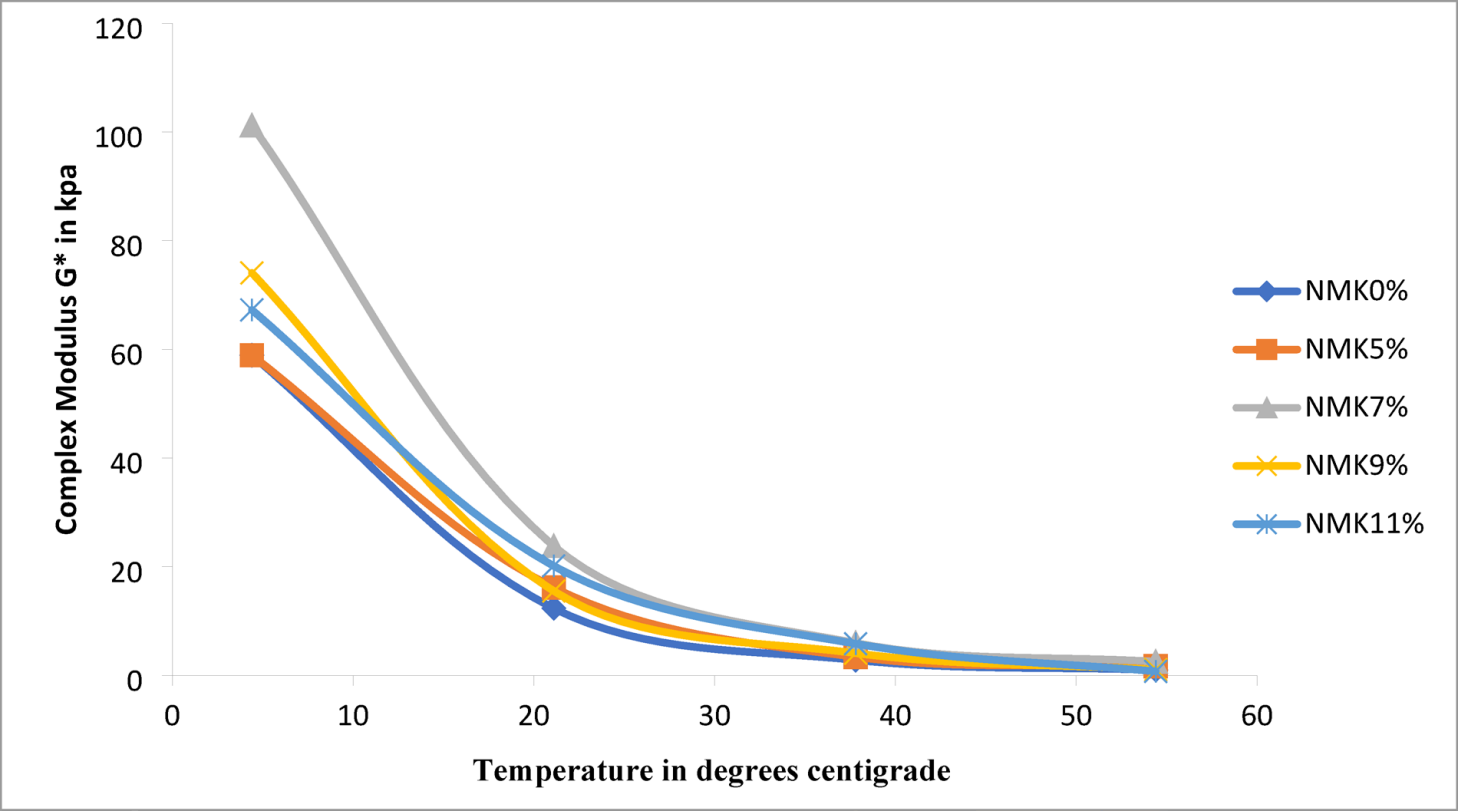
Relation between temperature and Complex modulus G* with different percentage of NMK additive.

This experiment was conducted on five asphalt binder samples modified with NMK at contents of 0, 5, 7, 9, and 11% by weight of asphalt. For each NMK content, two replicate samples were tested, and the reported results represent the average values used to determine the complex shear modulus (G*) and phase angle (δ). The testing was performed assuming linear viscoelastic behavior. The results of this experiment evaluate the effect of NMK addition on both unmodified and modified asphalt mixtures. The tests were conducted at temperatures of 4.4, 21.1, 37.8, and 54.4 °C and frequencies of 25, 10, 1, 0.5, and 0.1 Hz. The results are presented in Table 15 and illustrated in Fig. 15. The findings indicate that the addition of NMK enhances the rheological performance of asphalt mixtures, particularly at higher temperatures. The complex modulus (G* increases with NMK content up to 7%, indicating improved resistance to rutting. However, further increases in NMK content beyond 7% lead to a reduction in G* suggesting a decline in performance compared to the control mixture without NMK. This behavior implies that excessive NMK content may adversely affect fatigue and cracking resistance. Overall, the optimum NMK content was found to be 7%, which provided the best balance in improving the mechanical and rheological properties of the asphalt mixtures, as shown in Table 15 and Fig. 15.

**Table 15:** Dynamic modulus test for asphalt mixture.

NMK %		Zero%		5%		7%		9%		11%	
Temperature °C	Frequency, HZ	modulus G, MPa	Phase angle, Degree δ	modulus, G, MPa	Phase angle, Degree δ	modulus G, MPa	Phase angle, Degree δ	modulus, G, MPa	Phase angle, Degree δ	modulus G, MPa	Phase angle, Degree δ
4.4	25	9918	9.69°	132o7	10.38°	13,646	7.75	10,976	8.52	10,778	9.22
	10	9120	10.63°	12,076	11.33°	12,762	8.72	10,042	9.73	10,072	9.98
	5	8451	11.66°	11,156	12.53°	11,990	9.54	9311	10.69	9499	10.76
	1	6882	14.53°	8950	15.87°	10,160	11.75	7602	13.42	8056	13.02
	0.5	6189	15.90°	8041	17.43°	9346	12.91	6843	14.85	7499	14.06
	0.1	4692	19.90°	5954	21.88	7441	16.1	5205	18.61	6115	17.17
21.1	25	4482	21.25°	6288	22.87	7102	17.34	5236	19.66	6230	18
	10	3744	23.65°	5110	25.52	6066	19.42	4346	22.05	5458	19.9
	5	3209	25.49°	4271	27.59	5315	21.19	3722	23.98	4853	21.5
	1	2089	30.32°	2602	32.73	3708	25.82	2453	28.92	3481	26.03
	0.5	1734	31.63°	2070	33.87	3155	27.43	2037	30.3	3034	27.08
	0.1	9912	35.49°	1154	35.6	2018	31.73	1232	34.21	2055	30.98
37.8	25	1629	35.06°	2215	40.52	2900	29.88	2184	32.15	2857	29.28
	10	1244	35.77°	1658	39.09	2268	31.75	1674	33.67	2301	30.47
	5	979	36.23°	1249	38.29	1853	32.73	1341	34.4	1914	31.16
	1	529.6	36.47°	661.3	35.56	1076	34.61	762.9	35.31	1201	32.51
	0.5	418.4	35.16°	536.5	32.62	868.9	33.72	611	34.06	1009	31.13
	0.1	240.3	31.96°	360.2	26.77	516.4	31.77	371	31.67	669.2	28.77
54.4	25	589.9	35,70°	759.9	26.28	1263	29.83	651.4	36.6	568	34.48
	10	465.9	31.97°	668.2	22.25	996.5	27.6	510.4	33.15	420.6	33,96
	5	355.2	30.66°	579.6	20.19	827.9	25.81	387.4	31.92	224.7	31.71
	1	204.3	27.02°	460.4	15.61	592.9	21.81	229.1	27.15	186.3	29.32
	0.5	177.2	24.50°	428.7	13.56	536.4	19.67	196.6	24.15	125.6	25.47
	0.1	132.7	20,09°	273.2	8.36	445.3	16.7	146.6	18.88	568	34.48

## Conclusions

This study demonstrates the added value of integrating conventional tests with Superpave-based rheological analysis. By combining empirical performance measures with fundamental parameters such as the dynamic shear modulus (G*), a clearer mechanistic understanding of the influence of NMK on stiffness, deformation resistance, and cracking behavior is achieved. The main findings of the laboratory tests and the quantified benefits of modifying asphalt binders and mixtures with NMK can be summarized as follows:

1. The use of NMK as an asphalt modifier contributes to improved pavement performance, particularly in terms of extended service life, as indicated by laboratory evaluations of both asphalt binders and mixtures.
2. Compared to unmodified mixtures, NMK-modified mixtures exhibited higher Marshall stiffness modulus, indirect tensile strength, and rutting stiffness, indicating improved resistance to cracking and permanent deformation. Furthermore, increasing NMK content resulted in higher values of stability, air voids, and voids in mineral aggregate (VMA).
3. The results of the complex shear modulus test for asphalt mixtures showed that the addition of NMK increases the rutting resistance parameter, indicating improved resistance to rutting, permanent deformation, and cracking. The optimum NMK content for asphalt mixtures was found to be 7% by weight of asphalt.
4. Dynamic shear rheometer (DSR) test results indicated that NMK modification enhances rutting and cracking resistance of asphalt binders. The optimum NMK content for binders was determined to be 5% by weight of asphalt.
5. Overall, a dosage of 7% NMK by weight of asphalt provide the most significant improvements in pavement performance, including enhanced resistance to rutting, fatigue, and cracking.

In conclusion, the incorporation of NMK as a modifier in asphalt pavements can significantly enhance pavement performance and provides a practical approach for improving highway design and construction. This study is limited to laboratory conditions and specific materials, which may affect the generalization of results. Future work should include field studies, long-term performance evaluation, and investigation of different materials and NMK contents, along with economic and environmental assessments.

## Data Availability

All data generated or analysed during this study are included in this published article.
